# Restenosis Inhibition and Re-differentiation of TGFβ/Smad3-activated Smooth Muscle Cells by Resveratrol

**DOI:** 10.1038/srep41916

**Published:** 2017-02-06

**Authors:** Yichen Zhu, Toshio Takayama, Bowen Wang, Alycia Kent, Mengxue Zhang, Bernard Y.K. Binder, Go Urabe, Yatao Shi, Daniel DiRenzo, Shakti A. Goel, Yifan Zhou, Christopher Little, Drew A. Roenneburg, Xu Dong Shi, Lingjun Li, William L. Murphy, K. Craig Kent, Jianjuan Ke, Lian-Wang Guo

**Affiliations:** 1 Department of Surgery, University of Wisconsin, 5151 Wisconsin Institute for Medical Research, 1111 Highland Ave, Madison, WI 53705, USA; 2 Department of Urology, Capital Medical University Beijing Friendship Hospital, N0. 95, Yong’an Road, Xicheng district, Beijing, China; 3 University of Wisconsin Hospital and Clinics, 600 Highland Ave, Madison, WI 53792, USA; 4 Department of Biomedical Engineering, University of Wisconsin, 5009 Wisconsin Institute for Medical Research, 1111 Highland Ave, Madison, WI 53705, USA; 5 School of Pharmacy and Department of Chemistry, University of Wisconsin, Madison, WI 53705, USA; 6 School of Life Sciences, Tianjin University, No. 92 Weijin Road, Nankai District, Tianjin 300072, China; 7 Department of Anesthesiology, Medical College, Wuhan University, 169 Donghu Road, Wuhan, Hubei, PR China

## Abstract

To date, there is no *periadventitial* drug delivery method available in the clinic to prevent restenotic failure of open vascular reconstructions. Resveratrol is a promising anti-restenotic natural drug but subject to low bioavailability when systemically administered. In order to reconcile these two prominent issues, we tested effects of periadventitial delivery of resveratrol on all three major pro-restenotic pathologies including intimal hyperplasia (IH), endothelium impairment, and vessel shrinkage. In a rat carotid injury model, periadventitial delivery of resveratrol either via Pluronic gel (2-week), or polymer sheath (3-month), effectively reduced IH without causing endothelium impairment and vessel shrinkage. In an *in vitro* model, primary smooth muscle cells (SMCs) were stimulated with elevated transforming growth factor (TGFβ) and its signaling protein Smad3, known contributors to IH. TGFβ/Smad3 up-regulated Kruppel-like factor (KLF5) protein, and SMC de-differentiation which was reversed by KLF5 siRNA. Furthermore, TGFβ/Smad3-stimulated KLF5 production and SMC de-differentiation were blocked by resveratrol via its inhibition of the Akt-mTOR pathway. Concordantly, resveratrol attenuated Akt phosphorylation in injured arteries. Taken together, periadventitial delivery of resveratrol produces durable inhibition of all three pro-restenotic pathologies — a rare feat among existing anti-restenotic methods. Our study suggests a potential anti-restenotic modality of resveratrol application suitable for open surgery.

Post-operative vessel re-narrowing (or restenosis) occurs in a great number of patients undergoing vascular reconstructions because of intimal hyperplasia (IH) and/or constrictive vessel remodeling^1^. Currently the clinically used anti-restenotic drugs are rapamycin (or analogs) and paclitaxel. While these drugs delivered by endovascular stents are effective in retarding IH, there is evidence that they cause stent-edge constrictive remodeling^2^. Moreover, they delay the recovery of damaged endothelium, predisposing patients to in-stent thrombosis and sudden death^3^,^4^. Therefore, drugs/delivery methods that can mitigate IH without causing endothelium impairment and constrictive remodeling make attractive candidates for next-generation anti-restenotic therapy^5^. Resveratrol, a polyphenol enriched in grapes, has been shown to be somewhat beneficial in animal models of atherosclerosis and restenosis in several reports^6^,^7^,^8^,^9^,^10^,^11^. However, the efficacy is relatively low in these studies, presumably due to the use of systemic delivery approaches (intraperitoneal, intravenous, intra-gavage, or dietary). It is well known that the extremely low bioavailability of resveratrol poses a major limitation for its translation into clinical use^12^,^13^,^14^. Thus, local delivery methods, which hold promise for prolonging bioactivity of this drug, are being actively pursued^14^.

In contrast to systemic delivery, periadventitial administration prevents drug from directly entering circulation and hence its rapid metabolic degradation^15^,^16^. More importantly, a periadventitial approach is uniquely suited for over 300,000 patients (each year in the US alone) who undergo *open surgeries* including bypass, endarterectomy, and dialysis access^17^. Of particular note, currently there are no drug delivery methods available for these patients to prevent restenosis^15^. Drug-eluting stents are used for the management of restenosis following angioplasty, but this method cannot be applied to open surgeries^18^. Hence in this specific context, to evaluate the potential of resveratrol as a candidate anti-restenotic therapeutic for open surgery, we employed a periadventitial^15^, local (as opposed to systemic) delivery approach to determine its full-spectrum impact on the major pathological processes contributing to restenosis, including IH, endothelium damage, as well as constrictive remodeling. Periadventitial delivery of resveratrol has not been previously assessed as an alternative approach to improve its anti-restenotic efficacy.

Given the therapeutic potential of resveratrol for prevention of restenosis, it is important to understand its functional mechanisms. In previous studies using primary vascular SMCs treated with various stimuli, resveratrol was shown to inhibit cell proliferation and migration, both IH-promoting behaviors^5^,^19^. Most recently, Thompson *et al*. found that resveratrol inhibited platelet derived growth factor (PDGF-BB)-stimulated SMC de-differentiation^20^. It has been recognized that the phenotypic plasticity of vascular SMCs is key to understanding the pathological mechanisms of IH^19^. Following surgical damage of the endothelium and elastic laminas, SMCs in the media become exposed to a range of stimuli, particularly potent being PDGF and transforming growth factor (TGFβ)^19^,^21^. Activated SMCs then undergo de-differentiation, as manifested by reduced expression of contractile proteins^22^ including calponin, smooth muscle myosin heavy chain (SM-MHC), and smooth muscle actin (α-SMA)^20^. They migrate into the intima, proliferate, and form a highly cellular neointima layer intruding into the lumen (restenosis)^23^. Thus SMC de-differentiation is fundamental for the SMC transformation to pathogenic phenotypes (e.g. migration and proliferation)^24^. In this regard it is both important and advantageous to use resveratrol as a pharmacological probe to interrogate the molecular mechanisms of SMC de-differentiation and abrogation thereof (or re-differentiation). Although previous studies reveal that the anti-proliferative function of resveratrol in SMCs is related to anti-oxidant and anti-inflammatory effects^10^,^11^, the mechanism underlying resveratrol inhibition of SMC de-differentiation is relatively little understood. Thompson *et al*. found that at low and high concentrations resveratrol promotes SMC contractile phenotype by activating sirtuin1 and AMPK, respectively^20^. However, it remains unknown whether resveratrol re-differentiates SMCs by modulating transcription factors, which play a master role for IH-promoting SMC de-differentiation^25^.

The best-known transcription factors involved in SMC phenotype switching are KLF4 and KLF5^26^. Whereas the role of KLF4 is somewhat controversial, KLF5 has been consistently shown to promote SMC de-differentiation, migration and proliferation *in vitro*, all phenotypes contributing to IH. While KLF5 siRNA knockdown reduces, KLF5 overexpression enhances IH^27^. Up-regulation of KLF5 is found in human and rat restenotic lesions, and higher KLF5 levels are correlated with higher incidence of restenosis and cardiac allograft vasculopathy in human patients^28^. As such, KLF5 is an ideal transcription factor for our mechanistic study of SMC de-differentiation. We have previously found that TGFβ and its signaling protein Smad3 are both up-regulated in neointimal SMCs and contribute to IH^21^. Most recently, we further observed that TGFβ/Smad3 effected potent stimulation for SMC de-differentiation in cultured SMCs^22^. However, it is not known whether TGFβ/Smad3-stimulated SMC de-differentiation is related to KLF5.

In this study, we first evaluated the periadventitial delivery mode of resveratrol as a potentially viable method for restenosis prevention after open surgery, by systematically analyzing its effects on all three major pro-restenotic pathologies using a rat balloon injury model of restenosis. We found that the periadventitial approach had a markedly improved inhibitory effect on IH versus systemic delivery in previous reports. Remarkably, the recovery of damaged endothelium was not impaired but rather enhanced, and constrictive remodeling did not occur. Then using an established *in vitro* system we investigated the mechanism by which resveratrol re-differentiates rat aortic SMCs that are de-differentiated by elevated TGFβ/Smad3 signaling. We focused our mechanistic studies on SMC de-differentiation because it is fundamentally important for SMC pathogenic phenotype transformation and IH yet the molecular mechanism remains relatively poorly understood^22^,^25^. Moreover, it is of our particular interest to further investigate a novel finding from our latest report, i.e. TGFβ/Smad3 stimulates SMC de-differentiation^22^. Thus, it was based upon this specific context that we determined the mechanism of re-differentiation of TGFβ/Smad3-activated SMCs by resveratrol. We found that TGFβ/Smad3 treatment de-differentiated SMCs via up-regulation of KLF5 protein, and resveratrol could re-differentiate those cells by blocking KLF5 up-regulation. Ultimately, our results in this study suggest that when periadventitially delivered, resveratrol is a favorable anti-restenotic agent with significant potential clinic utility.

## Methods

### Animals

All animal procedures conformed to the NIH guide for the ethical care and use of laboratory animals. Animal protocols were approved by the Institutional Animal Care and Use Committee of University of Wisconsin-Madison. All surgeries were performed under isoflurane anesthesia (inhalation at 2 ml/min flow rate), and every effort was made to minimize animal suffering. Animals were euthanized in a chamber gradually filled with CO_2_.

### Rat carotid artery balloon angioplasty model

Carotid artery balloon angioplasty was performed in male Sprague-Dawley rats (Charles River, 300–350 g) as previously described^29^. Briefly, rats were anesthetized by inhalation of 2.5% isoflurane (throughout this study). The left common carotid artery was exposed through a midline cervical incision. A 2 F Fogarty catheter (Edwards Lifesciences) was inserted into the common carotid artery *via* an arteriotomy in the external carotid artery. To produce arterial injury, the balloon was inflated and withdrew to the carotid bifurcation and this action was repeated three times. The external carotid artery was then permanently ligated, and blood flow was resumed.

### Periadventitial local delivery of resveratrol to injured arteries

For short-term periadventitial resveratrol delivery, we applied a widely used Pluronic gel method as described in our previous report^5^,^29^. Briefly, to ensure complete solubility, 500 μg of resveratrol (Sigma-Aldrich) from a DMSO stock was first dissolved in 30 μl of 10% DMSO and then mixed with 270 μl of 25% F-127 Pluronic gel (Sigma-Aldrich) that was kept on ice. Immediately after balloon angioplasty in the left common carotid artery, the gel containing resveratrol was applied to the outside of the injured segment of the carotid artery and solidified right away. In the control group, equal volume of vehicle (30 μl of 20% DMSO) mixed with Pluronic gel was applied. Since Pluronic gel dissolves within 3 days^16^, for long-term periadventitial delivery we used a poly(ε-caprolactone) (PCL) sheath approach that was established by our groups and able to produce steady release for at least 2 months. Briefly, resveratrol-loaded PCL sheaths (100 μg per sheath) were prepared using our solvent casting method^18^. Control sheaths were prepared using the same procedures but with no resveratrol added. Immediately after balloon injury, a PCL sheath was longitudinally placed onto the injured artery segment. It was wrapped in such a way that only ~90% of the outer surface of the vessel was covered so as to avoid constriction of normal blood flow. The neck incision was then closed using a suture and animals were kept on a 37 °C warm pad for recovery. To avoid resveratrol photoisomerization, aliquots of the stock solution were stored in a sealed Eppendorf tube wrapped in aluminum foil, and experiments involving resveratrol were kept from constant light exposure.

### Mass spectrometry analysis of resveratrol administered into the arterial wall

In order to detect the molecules of resveratrol (and/or its metabolites) that penetrated into the arterial wall, we used a mass spectrometry (MS)-based strategy following our published method^30^,^31^.

#### Sample preparation

We collected (1 day after surgery) the injured rat carotid arteries that were treated with resveratrol-containing periadventitial Pluronic gel, and immediately snap-froze the samples in liquid N_2_. For resveratrol extraction, 500 μl MeOH/H_2_O (80:20, V/V) was added to ground tissue homogenates, incubated for 30 min at 60 °C on shaking, and then centrifuged for 5 min at 10,000 rcf. The supernatant was transferred to a new Eppendorf tube and dried with Speed-Vac. The tissue extract was finally dissolved in 100 μl MeOH/H_2_O (80:20, V/V) and a 5 ul aliquot was injected for MS analysis.

#### Mass spectrometry

The MS analysis was performed using an ultra-performance liquid chromatography system equipped with a Q-Exactive Hybrid Quadrupole-Orbitrap mass spectrometer in positive ion mode. A reversed phase C18 column was employed. Mobile phases: 0.1% FA/H_2_O (A) and 0.1% FA/ACN (B). LC gradient: 0–5 min, 0–50% B; 5–7 min, 50–100% B; 7–8 min, 100 % B; 8–9 min, 100–0% B; 9–12 min, 0% B. The flow rate was 0.3 ml/min and column temperature was 30 °C. The injection volume for each run was 2 μl. MS experiments were performed with a resolution of 70,000 (at m/z 400), a mass range of m/z 90–1000, a maximum injection time of 100 ms, and 2 microscans per scan. The spray voltage was 3.5 kV and the sheath gas flow was 48 psi. A standard sample of resveratrol was used for reference. A series of resveratrol standard solutions were prepared at concentrations of 1 ng/μl, 0.5 ng/μl, 0.1 ng/μl and 0.01 ng/μl. Each 5 μl standard solution was injected into the mass spectrometer.

### Morphometric analysis of intimal hyperplasia, vessel remodeling, and restenosis

Two weeks (Pluronic gel delivery) or three months (PCL sheath delivery) after balloon angioplasty, common carotid arteries were collected from anesthetized animals (under 2.5% isoflurane) following perfusion fixation at a physiological pressure of 100 mmHg^29^, the animals were then euthanized in a CO_2_ chamber. Paraffin sections (5 μm thick) were excised at equally spaced intervals and eosin or van Gieson-stained for morphometric analysis, as described in our previous reports^29^. Planimetric parameters as follows were measured on the sections and calculated using Image J: the area inside external elastic lamina (EEL area) or internal elastic lamina (IEL area), lumen area, intima area (=IEL area − lumen area), and media area (=EEL area − IEL area). Measurements were performed by a student blinded to the experimental conditions using 3–5 sections from each of 4–6 rats treated with either vehicle (DMSO) or resveratrol. The data from all sections were pooled to generate the mean for each animal. The means from all the animals in each treatment group were then averaged, and the standard error of the mean (SEM) was calculated. The vessel size is defined by EEL length; intimal hyperplasia is quantified as a ratio of intima area versus media area.

### Immunohistochemistry for assessment of phospho-Akt or caspase-3 positive cells in the rat arterial wall

Immunostaining was performed on rat carotid artery sections following our published methods^5^,^29^. Briefly, the sections were first incubated with each of the primary antibodies: anti- caspase-3 (Cell Signaling, Cat#9661), 1:500 dilution, incubated for 1 h; anti-p-AKT (Cell Signaling, Cat#4060, clone D9E), 1:40 dilution, incubated for overnight at 4 °C. The sections were then incubated with the ImmPRESS HRP-conjugated goat-anti-rabbit secondary antibody (Vector Laboratories, 1:200), followed by visualization with 3, 3-diaminobenzidine (DAB). The slides were counterstained with hematoxylin. The number of positively stained cells was counted on 8-bit binary images converted by Image J from the pictures of immunostained sections and normalized by the high power microscopic field (HPF). Cell counting was performed by a student blinded to experimental conditions. In each experimental group (DMSO control or resveratrol treatment), at least 5 sections from each of 4–6 animals were used. The data from all sections were pooled to generate the mean for each animal. The means from all animals in each experimental group were then averaged, and the standard error of the mean (SEM) was calculated.

### Assessment of post-angioplasty re-endothelialization

Re-endothelialization in balloon-injured arteries was evaluated on day 14 after angioplasty by CD31 immunostaining using the method described above^5^. Briefly, a goat anti-CD31 primary antibody (R&D Sytems,1:150) was incubated with the sections for 1 h followed by an incubation with a biotinylated rabbit-anti-goat secondary antibody for 30 min. Immunostaining of CD31 was then visualized by using streptavidin-HRP and DAB. For quantification of re-endothelialization, we used Image J to measure the percentage of CD31-stained versus total peri-luminal perimeter^5^,^16^.

### *In vitro* stimulation of primary SMCs and treatment with resveratrol

Rat aortic vascular SMCs were isolated from thoracoabdominal aortas of male Sprague–Dawley rats and maintained at 37 °C and 5% CO_2_ in low glucose Dulbecco’s Modified Eagle Medium (DMEM) (Gibco, Carlsbad, CA) supplemented with 10% fetal bovine solution (FBS)^5^. Cells were used at passages 5–6 for all experiments. Cell viability was >95% as indicated by Trypan Blue exclusion assay. For stimulation of SMCs with elevated TGFβ/Smad3 signaling, adenoviral vectors expressing Smad3 (AdSmad3) and GFP (AdGFP control) were constructed as previously described^22^,^32^. SMCs were infected for 4 h with AdSmad3 (or AdGFP) (3 × 10^4^ particles/cell) in DMEM containing 2% FBS, and recovered for 20 h with 10% FBS, and then starved with 0.5% FBS for 24 h followed by treatment with human recombinant TGFβ1 (5 ng/ml, R&D Systems, Minneapolis, MN) or equivalent amount of solvent (final 4 μM HCl and 1 μg/ml BSA) for the time length indicated in figure legends. Cells were then harvested for various assays. For treatment with small molecular inhibitors, cells were pre-incubated for 2 h with an inhibitor at desired concentrations or with equal volume of DMSO (vehicle control) before TGFβ1 was added. To illustrate SMC morphology, Calcein (2 μM, Fisher Scientific) was incubated with the cell culture for 30 min at 37 °C, and cells were washed 3× with PBS prior to fluorescence microscopy followed by cell area calculation using Image J.

### siRNA knockdown of KLF5

SMCs were infected with AdGFP or AdSmad3 as described above. Prior to treatment with TGFβ1, knockdown of KLF5 was performed using siRNA oligos according to manufacturer’s protocols. Briefly, SMCs of 60–70% confluence were transfected with 20 nM KLF5-specific siRNA (Silencer siRNA, s136575) or scrambled control siRNA (Silencer siRNA, AM4611) (Ambion, Carlsbad, CA) for 6 h using Lipofectamine RNAiMAX (13778, Invitrogen, Carlsbad, CA) in OptiMEM I medium containing 0.5% FBS but no antibiotics.

### Quantitative real-time PCR (qRT-PCR)

mRNA was isolated from collected cells using Trizol (Qiagen, Valencia, CA) following the manufacturer’s instructions. Purified mRNA (1 μg) was used for the first-strand cDNA synthesis using iScript cDNA synthesis kit (Bio-Rad) and quantitative RT-PCR was performed using the 7500 Fast Real-Time PCR System (Applied Biosystems, Carlsbad, CA). Each cDNA template was amplified in triplicates using SYBR Green PCR Master Mix (Applied Biosystems, Carlsbad, CA) with primers for rat CD34 or rat KLF5.

### Western blotting for evaluation of protein levels

SMCs were lysed in RIPA buffer containing protease inhibitors (50 mM Tris, 150 mM NaCl, 1% Nonidet P-40, 0.1% sodium dodecyl sulfate, and 10 μg/ml aprotinin). Protein concentrations of cell lysates were determined using a Bio-Rad DC™ Protein Assay kit. Approximately 15~30 μg of proteins from each sample were separated on 4–20% Mini-PROTEAN TGX precast gels (Bio-Rad) and transferred to a PVDF membrane. Proteins of interest were detected by immunoblotting using the following primary antibodies and dilution ratios: anti-calponin (1:1000) from Santa Cruz (sc-58707), anti-SM-MHC (1:1000) from Santa Cruz (sc-6956), anti-KLF5 (1:250) from Sigma-Aldrich, anti-pmTOR (1:1000) from Cell Signaling (5536 S), anti-total mTOR (1:1000) from Cell Signaling (2972), anti-pS6K (1:1000) from Cell Signaling (9204 S), anti-total S6K (1:1000) from Cell Signaling (2708 S), anti-pAkt (1:1000) from Cell Signaling (9271 S), anti-total Akt (1:1000) from Cell Signaling (4691 S), anti-pSmad3 (1:1000) from Invitrogen (44246 G), anti-total Smad3 (1:1000) from Invitrogen (511500) and mouse anti-β-actin from Sigma-Aldrich. After incubation of the blots with HRP-conjugated secondary antibodies (1:3000 for goat anti-rabbit or 1:10000 for goat anti-mouse, Bio-Rad), specific protein bands on the blots were visualized by applying enhanced chemiluminescence reagents (Pierce) and then recorded with a LAS-4000 Mini imager (GE, Piscataway NJ). Band intensity was quantified using ImageJ.

### Statistical analysis

Data are presented as means ± SEM. Means were compared between two groups using two-tailed unpaired Student’s t-test. For multiple comparisons the analysis of variance (ANOVA) test followed by Bonferroni’s correction was applied. A value of *P* < 0.05 was considered significant. All *P-*values are two-sided. Statistics were performed using the GraphPad prism 6.0 Software (GraphPad Software, Inc., La Jolla, CA, USA).

## Results

### Periadventitial delivery of resveratrol eliminates neointima without causing constrictive remodeling two weeks after balloon injury of rat carotid arteries

In several reports resveratrol was shown to suppress IH but with limited efficacy (~30–60% inhibition)^6^,^7^,^8^,^9^,^10^,^11^. It is notable that in these studies resveratrol was administered via systemic approaches, which might have led to its quick destruction by metabolic systems. To circumvent this problem, here we adopted a periadventitial delivery approach so that resveratrol could evade circulation and rapid systemic clearance.

We performed rat carotid balloon angioplasty, a widely accepted model of IH^18^, and then applied resveratrol in Pluronic gel around the injured artery. We confirmed that the resveratrol compound was able to penetrate into the arterial wall, as indicated by mass spectrometry analysis using artery tissue homogenates (Figure S1). At 24 h after periadventitial application, resveratrol was present in the injured rat carotid arteries in two major forms, non-metabolized resveratrol and its monoglucuronide conjugate (Figure S2), with a ratio of the latter versus the former at 28.4% ± 17.3 (standard error of the mean, n = 3 rats). This data indicates that majority of resveratrol molecules in the vessel wall were non-metabolized one day after periadventitial administration.

**Figure 1:**
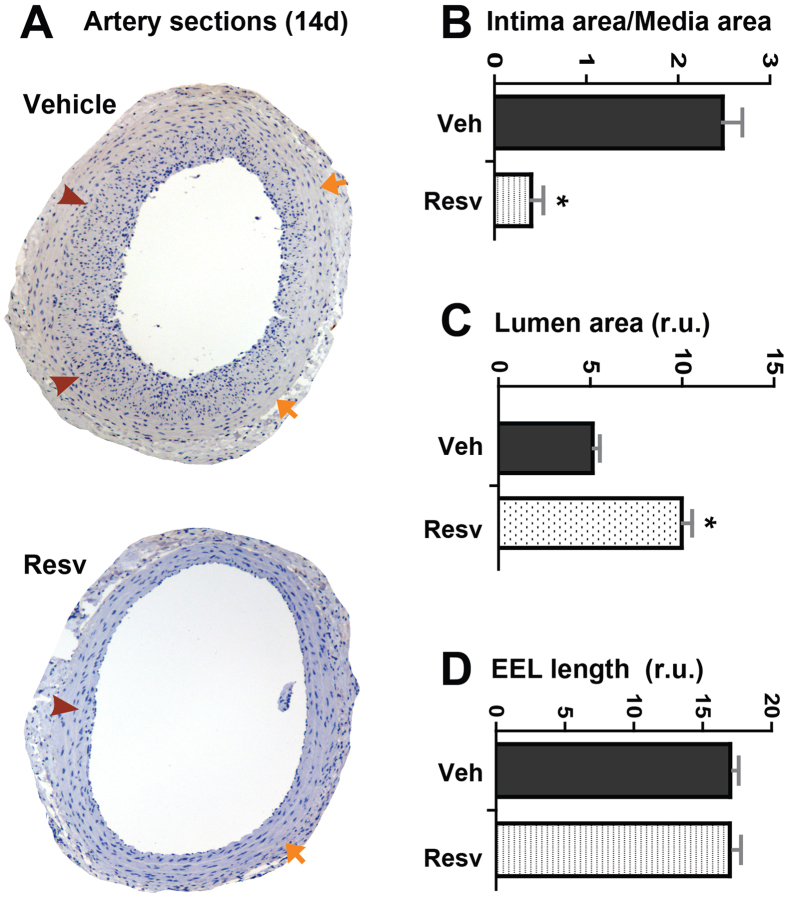
Effect of two-week resveratrol treatment on neointima formation, vessel size, and lumen size of rat carotid arteries following balloon injury. (**A**) Representative stained sections from arteries treated with vehicle (DMSO) and resveratrol respectively and collected on day 14 after injury. Arrows indicate external elastic lamina (EEL); arrowheads mark internal elastic lamina (IEL). (**B**–**D**) Quantified data of IH (ratio of intima/media area), lumen area, and arterial remodeling (EEL length), respectively. Each bar represents a mean (±SEM) of sections from 5 animals, *P < 0.05 compared to vehicle control.

We then evaluated the effect of resveratrol on IH. At day 14 after balloon injury and resveratrol administration, carotid arteries were collected and cross sections prepared. Morphometric analyses (Fig. 1) indicate that treatment with resveratrol resulted in a pronounced 86% decrease of IH (intima/media ratio) compared to vehicle (DMSO) control. The EEL length, which measures the overall vessel size (or remodeling), was unaltered. The ultimate outcome of resveratrol treatment is an approximately 2 fold increase of lumen area. These results demonstrate that periadventitial delivery of resveratrol is highly efficacious versus previously reported systemic delivery (~30–60% inhibition of IH)^6^,^7^,^8^,^9^,^10^,^11^.

### Periadventitial delivery of resveratrol enhances re-endothelialization two weeks after balloon injury of rat carotid arteries

**Figure 2:**
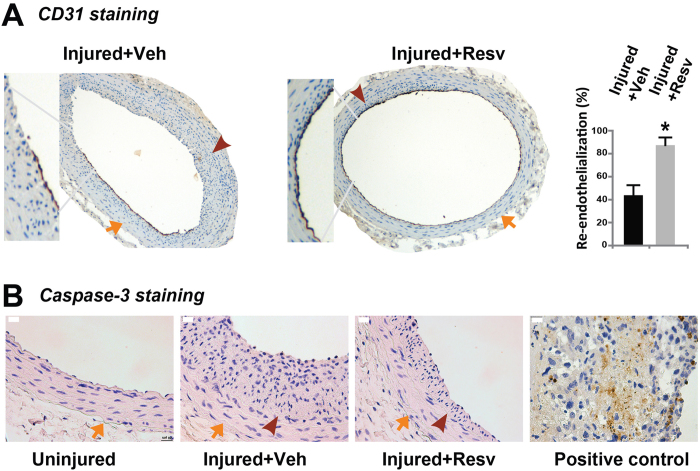
Effect of resveratrol treatment on re-endothelialization and cell apoptosis of rat carotid arteries after balloon injury. Rat carotid arteries were treated with either vehicle or resveratrol and collected on day 14 after injury. Arrows indicate external elastic lamina (EEL); arrowheads mark internal elastic lamina (IEL). (**A**) Immunostaining of CD31. Quantification: mean ± SEM; n = 3–5 animals; *P < 0.05 compared to vehicle control. (**B**) Immunostaining of cleaved caspase-3. Scale bar = 20 μm. A placenta sample serves as positive control.

Current clinical anti-restenotic managements (stents releasing rapalogs or paclitaxel) impair endothelium recovery following surgical damage^4^. As such these methods are associated with a high risk of in-stent thrombosis, a condition associated with ~50% mortality^3^. We therefore evaluated whether resveratrol delays post angioplasty re-endothelialization, by immunostaining CD31, a commonly used marker for vascular endothelium. Remarkably, statistical analysis of CD31 staining indicates that compared to vehicle control resveratrol treatment did not impair endothelium at two weeks after periadventitial administration, but rather, increased re-endothelialization by 2 fold (Fig. 2A). This result reveals an interesting protective effect of periadventitially delivered resveratrol on the vascular endothelium *in vivo*. Furthermore, a lack of apoptotic cells, which would otherwise be positively stained for cleaved caspase-3, indicates a healthy vessel after resveratrol treatment (Fig. 2B). The caspase-3 immunostaining method was validated by a positive control sample.

### Periadventitial delivery of resveratrol mitigates neointima without causing constrictive remodeling three months after balloon injury of rat carotid arteries

**Figure 3:**
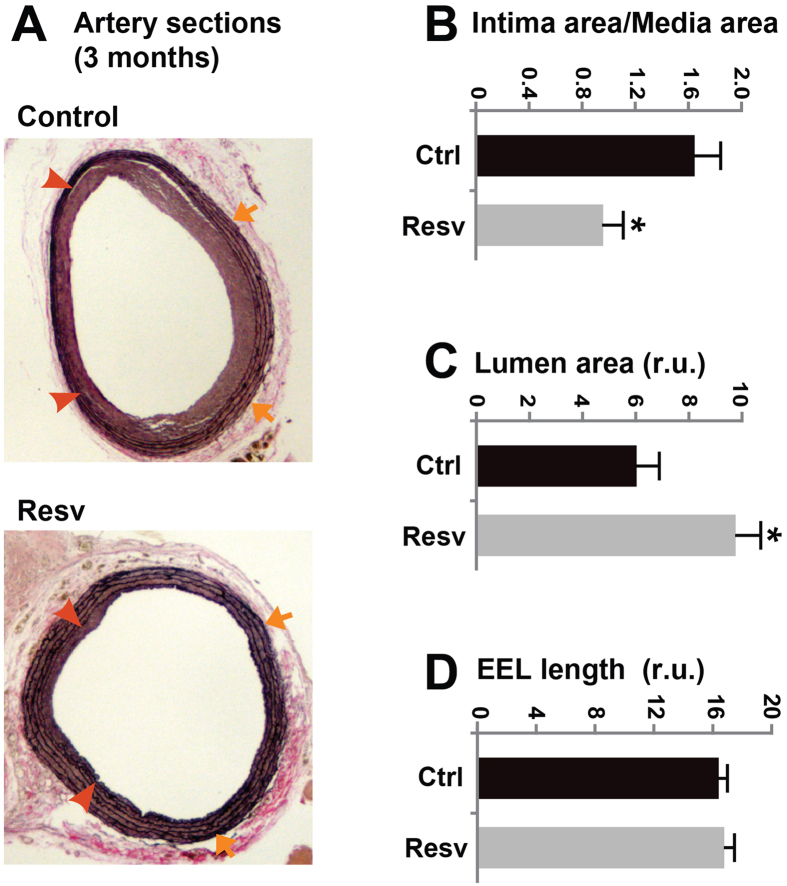
Effect of three-month resveratrol treatment on neointima formation, vessel size, and lumen size of rat carotid arteries following balloon injury. (**A**) Representative van Gieson-stained sections from arteries treated respectively with control PCL sheaths (no resveratrol) and resveratrol-loaded sheaths and collected at 3 months after injury. Arrows indicate external elastic lamina (EEL); arrowheads mark internal elastic lamina (IEL). (**B**–**D**) Quantified data of IH (ratio of intima/media area), lumen area, and arterial remodeling (EEL length), respectively. Each bar represents a mean (±SEM) of sections from 4–5 animals, *P < 0.05 compared to vehicle control.

Studies testing long-term effect of drug delivery have been rare presumably owing to a lack of suitable drug-releasing platforms as well as the difficulty of carrying out such experiments. We have recently developed a biodegradable PCL sheath that enables steady release of a hydrophobic compound (e.g. rapamycin) for at least 2 months^18^. Taking advantage of this technology, we produced resveratrol-loaded PCL sheaths and deployed them around injured segments of rat carotid arteries. After 3 months arteries were collected. Morphometric analyses indicate that IH was reduced by 42% in resveratrol-treated arteries compared to those treated with control PCL sheaths without resveratrol (Fig. 3). There was no significant change of EEL length. As a result, the lumen area was increased by 162%. These results indicate that given an appropriate periadventitial delivery method resveratrol can produce prolonged inhibitory effect on IH without causing constrictive remodeling.

### Resveratrol reverses TGFβ/Smad3-stimulated de-differentiation of cultured SMCs

Following surgical damage of the integrity of the endothelium and vascular wall, SMCs are exposed to various stimuli (e.g. growth factors) and undergo de-differentiation losing their signature contractile phenotype. This SMC phenotype change is critical for neointima formation^19^,^25^. Our group has previously shown that TGFβ and its signaling effector Smad3 are up-regulated following arterial injury and this signaling axis is an important stimulator of neointima formation^21^. Recently we observed that elevated TGFβ/Smad3 stimulate SMC de-differentiation^22^. Whether resveratrol inhibits TGFβ/Smad3-stimulated SMC de-differentiation is not known.

**Figure 4:**
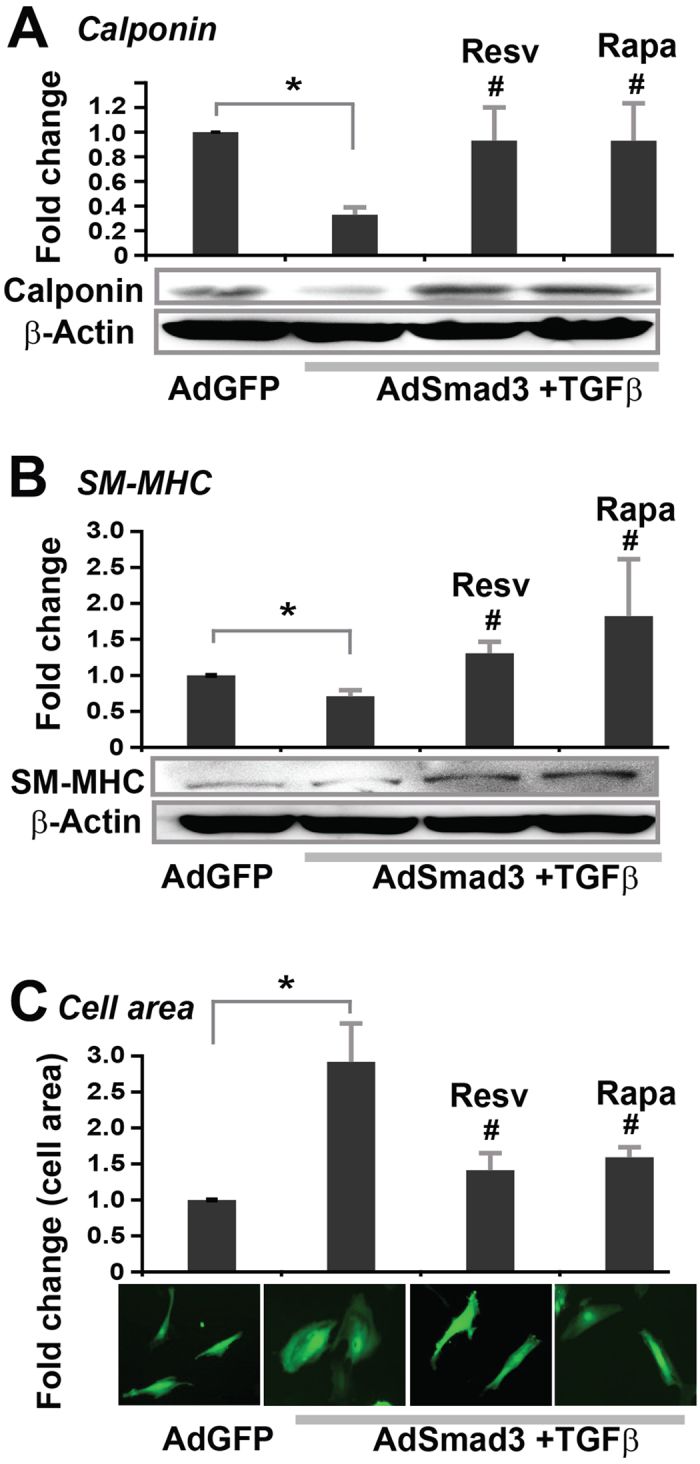
Inhibitory effect of resveratrol on TGFβ/Smad3-stimulated SMC de-differentiation *in vitro*. Rat aortic SMCs were infected with AdGFP (control) or AdSmad3 followed by pre-incubation with vehicle (DMSO) or resveratrol (50 μM) or rapamycin (200 nM) for 2 h, and then treated with solvent (for AdGFP control) or TGFβ1 (5 ng/mL) for 24 h. Cells were then used for Western blotting of calponin (**A**) or SM-MHC (**B**), or staining with Calcein for cell area measurement (**C**). Quantification: mean ± SEM of three independent experiments; normalization to β-actin; *P < 0.05 compared to AdGFP control; ^#^P < 0.05 compared to AdSmad3 + TGFβ1 without pre-incubation with a drug.

To mimic post-injury TGFβ/Smad3 up-regulation, we first expressed Smad3 (or GFP control) in rat primary aortic SMCs using adenovirus, and then added recombinant TGFβ1 (5 ng/ml) to activate Smad3 signaling^22^,^33^. Resveratrol (or DMSO control) was pre-incubated with the SMC culture for 2 h prior to TGFβ1 stimulation. We used resveratrol at 50 μM, an effective concentration without triggering apoptosis, as reported by Brito *et al*. using rat primary SMCs^34^. We defined SMC de-differentiation by changes in cellular morphology and in the expression of calponin and SM-MHC, the two most stringent vascular SMC markers^20^,^25^. As shown in Fig. 4, while TGFβ/Smad3 treatment markedly down-regulated calponin and SM-MHC as compared to AdGFP control, resveratrol completely reversed this change. Accordingly, while under TGFβ/Smad3 stimulation SMCs lost their characteristic spindle shape and transformed to an expanded morphology (quantified as increase of cell-occupied area) indicative of de-differentiation, treatment with resveratrol restored SMC spindle-shape morphology. Since it is known that rapamycin inhibits SMC de-differentiation^19^, we used rapamycin as a positive control and observed an effect similar to that of resveratrol. Together these results demonstrate that resveratrol can re-differentiate SMCs that are de-differentiated due to elevated TGFβ/Smad3 signaling.

### Resveratrol abrogates TGFβ/Smad3-stimulated KLF5 protein up-regulation in cultured SMCs

**Figure 5:**
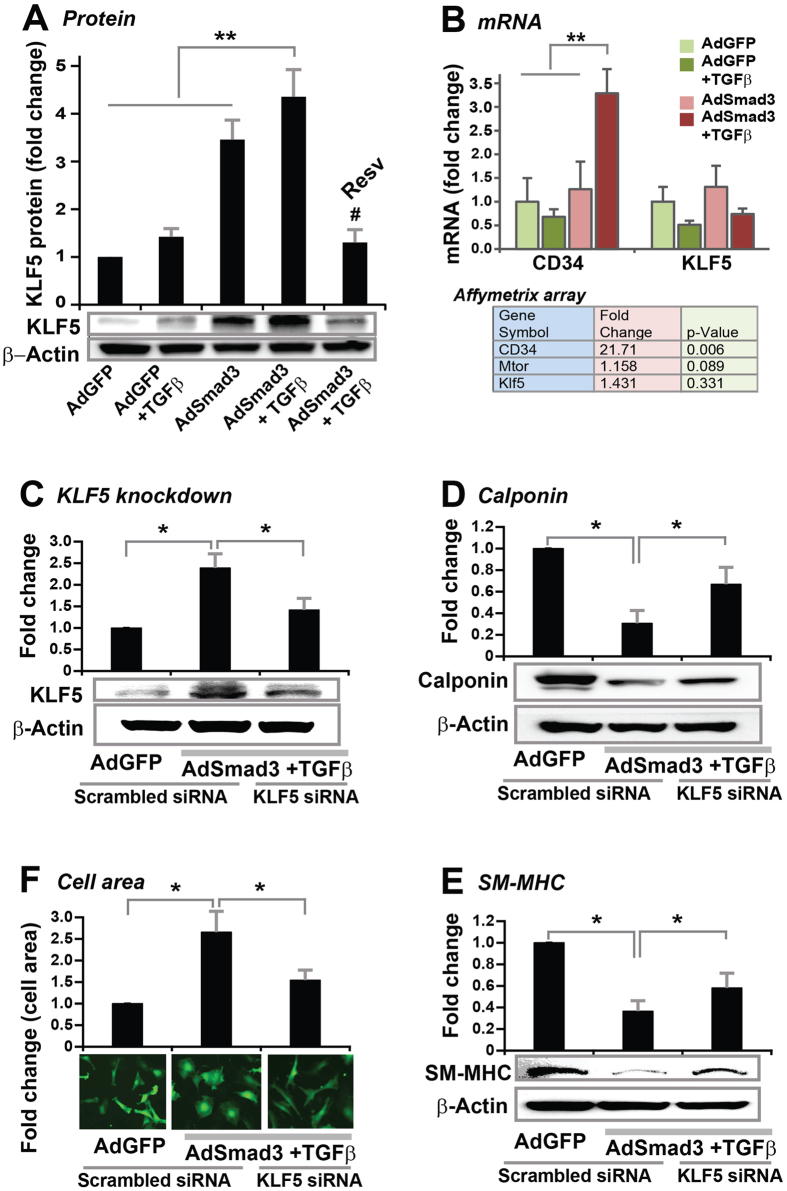
Inhibitory effect of resveratrol on TGFβ/Smad3-stimulated KLF5 protein production *in vitro*. Rat aortic SMCs were infected with AdGFP (control) or AdSmad3 followed by pre-incubation with vehicle (DMSO) or resveratrol (50 μM) for 2 h, and then treated with solvent or TGFβ1 (5 ng/mL) for 24 h. Cells were then collected for Western blotting analysis (**A**) or RT-PCR (**B**). Microarray data were retrieved from our recent report^22^. Quantification: mean ± SEM of three independent experiments; normalization to β-actin; **P < 0.05 compared to any of the first 3 conditions; ^#^P < 0.05 compared to AdSmad3 + TGFβ1 without pre-incubation with resveratrol. To determine the effect of KLF5 knockdown on TGFβ/Smad3-stimulated SMC de-differentiation, rat aortic SMCs were infected with AdGFP (control) or AdSmad3 and then transfected with scrambled or KLF5-specific siRNA for 6 h followed by treatment with solvent (control) or TGFβ1 (5 ng/mL) for 24 h. Cells were then used for Western blotting of KLF5 (**C**), calponin (**D**) or SM-MHC (**E**), or staining with Calcein for cell area measurement (**F**). Quantification: mean ± SEM of three independent experiments; normalization to β-actin; *P < 0.05 compared to AdSmad3 + TGFβ1 with scrambled siRNA (the middle bar).

We then further investigated the molecular mechanism underlying the resveratrol inhibitory effect on TGFβ/Smad3-stumulated SMC de-differentiation. SMC de-differentiation is known to be a transcriptionally regulated process but the molecular mechanisms remain to be better understood^25^. We first investigated KLF4 and KLF5, two prominent transcription factors involved in SMC de-differentiation^28^,^35^. We did not detect a significant change of KLF4 protein following TGFβ/Smad3 stimulation (data not shown). Interestingly however, through Western blotting, we found that either TGFβ or AdSmad3 alone increased KLF5 protein, and the combination of these two resulted in a 4-fold increase of KLF5 versus AdGFP control (Fig. 5A). We did not find a significant change of KLF5 mRNA by RT-PCR (Fig. 5B), which agrees with the Affymetrix result derived from our recent study^22^. These results reveal a novel finding that TGFβ/Smad3 up-regulate KLF5 protein (but not mRNA). Remarkably, resveratrol reversed this up-regulation by reducing KLF5 protein to the basal level (AdGFP control).

In order to determine a specific role of KLF5 in mediating TGFβ/Smad3-stimulated SMC de-differentiation, we used siRNA to knock down KLF5 in SMCs. As shown in Fig. 5C, in comparison to scrambled siRNA, KLF5-specific siRNA effectively reduced TGFβ/Smad3-stimulated KLF5 protein production. Consequently, KLF5 siRNA substantially restored protein levels of SMC markers calponin and SM-MHC that were down-regulated by TGFβ/Smad3 treatment (Fig. 5D and E). SMC re-differentiation due to specific KLF5 knockdown was accordantly reflected from SMC morphological changes; i.e. TGFβ/Smad3-transformed SMCs were reverted to a differentiated spindle shape by KLF5 siRNA (Fig. 5F). Thus, these results indicate that TGFβ/Smad3 initiate SMC de-differentiation in a KLF5-dependent manner.

### Resveratrol blocks TGFβ/Smad3-stimulated mTOR activation in cultured SMCs

**Figure 6:**
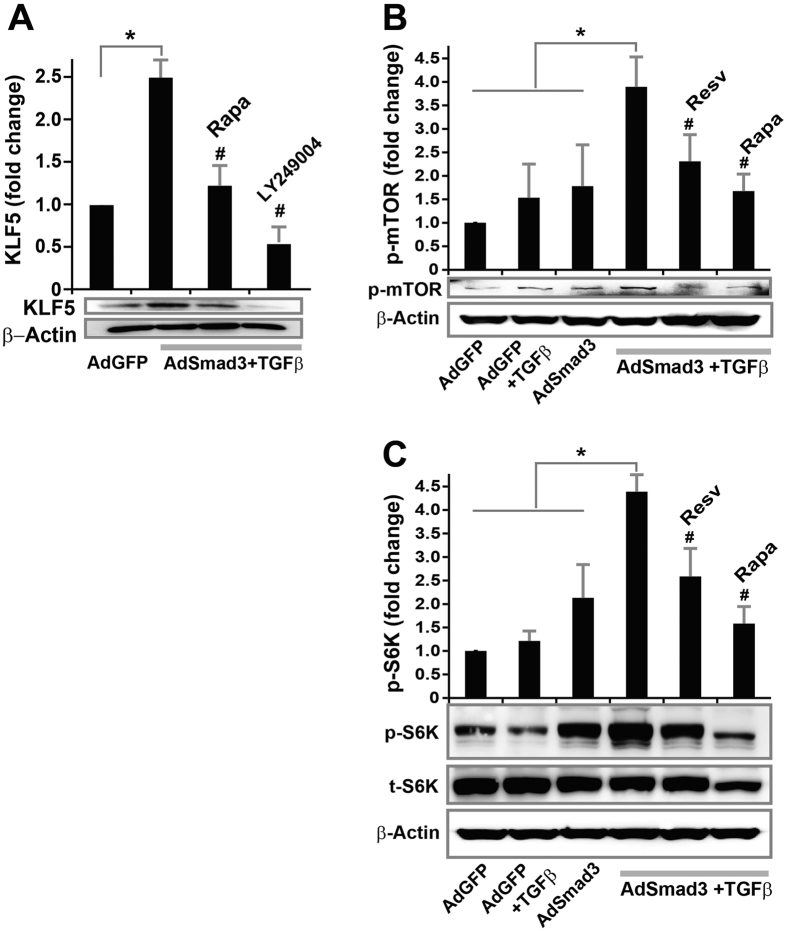
Inhibitory effect of resveratrol on TGFβ/Smad3-stimulated mTOR activation *in vitro*. Rat aortic SMCs were infected with AdGFP (control) or AdSmad3 followed by pre-incubation with vehicle (DMSO), resveratrol (50 μM), rapamycin (200 nM), or LY249004 (20 μM) for 2 h, and then treated with solvent or TGFβ1 (5 ng/mL) for 24 h. Cells were then collected for Western blotting analysis of KLF5 (**A**), p-mTOR (**B**) or p-S6K (**C**). Quantification: mean ± SEM of three independent experiments; normalization to β-actin or to total protein; *P < 0.05 compared to any of the first 3 conditions; ^#^P < 0.05 compared to AdSmad3 + TGFβ1 without pre-incubation with a drug.

For identification of upstream regulators responsible for KLF5 up-regulation, we noticed that similar to resveratrol, rapamycin was also able to re-differentiate TGFβ/Smad3-activated SMCs back to a contractile phenotype (Fig. 4). We thus determined the effect of rapamycin on KLF5 expression, and found that rapamycin at 200 nM (an effective concentration used in our previous report^36^) reversed TGFβ/Smad3-stimulated KLF5 protein up-regulation (Fig. 6A). As rapamycin is a specific mTOR inhibitor^19^, it is conceivable that elevated TGFβ/Smad3 signaling may activate the mTOR pathway in SMCs, which has never been previously reported. Indeed, we found that while TGFβ and Smad3 each increased p-mTOR, treatment with both stimulated mTOR phosphorylation nearly 4 fold (Fig. 6B). Interestingly, resveratrol substantially mitigated TGFβ/Smad3-stimulated mTOR phosphorylation. The assay was validated by the effect of rapamycin in blocking TGFβ/Smad3-stimulated mTOR phosphorylation. Furthermore, the same pattern of TGFβ/Smad3 stimulation and resveratrol inhibition was also observed with phosphorylation of S6K (Fig. 6C), the downstream effector and a frequently used surrogate of mTOR activation. This result confirms the TGFβ/Smad3 stimulatory effect on mTOR activation and its blockade by resveratrol.

### Resveratrol inhibits TGFβ/Smad3-stimulated Akt phosphorylation with no effect on Smad3 phosphorylation in cultured SMCs

**Figure 7:**
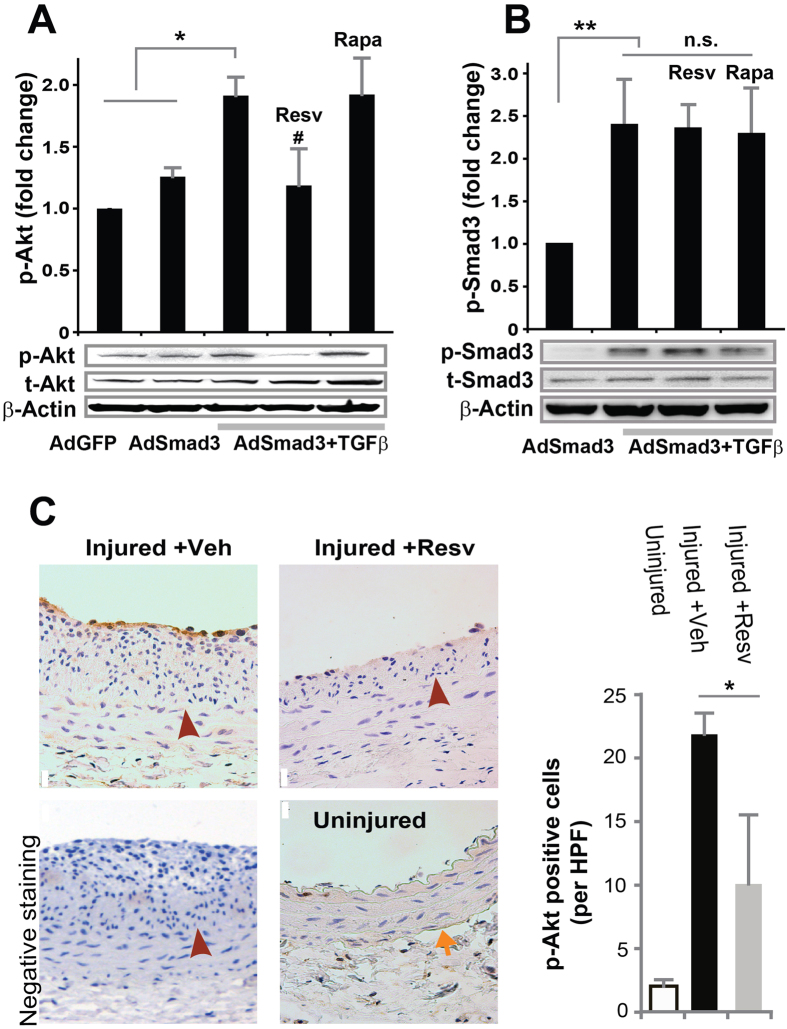
Inhibitory effect of resveratrol on TGFβ/Smad3-stimulated Akt activation *in vitro* and *in vivo* in balloon-injured rat carotid arteries. (**A**,**B**) Rat aortic SMCs were infected with AdGFP (control) or AdSmad3 followed by pre-incubation with vehicle (DMSO), resveratrol (50 μM), or rapamycin (200 nM) for 2 h, and then treated with solvent or TGFβ1 (5 ng/mL). For Western blotting analyses of p-Akt (**A**) and p-Smad3 (**B**), cells were collected after TGFβ1 treatment for 2 h and 0.5 h, respectively. Quantification: mean ± SEM of three independent experiments; normalization to total Akt (t-Akt) or to total Smad3 (t-Smad3); *P < 0.05 compared to each of the first 2 conditions; **P < 0.05 compared to AdSmad3 only; ^#^P < 0.05 compared to AdSmad3 + TGFβ1 without pre-incubation with a drug; n.s., not significant. (**C**). Rat carotid arteries were treated with either vehicle or resveratrol and collected on day 14 after angioplasty. Shown on the left are representative sections of p-Akt Immunostaining. Arrows indicate external elastic lamina (EEL); arrowheads mark internal elastic lamina (IEL). Scale bar = 20 μm. Negative staining: IgG instead of a primary antibody was used. Quantification: mean ± SEM of 4–5 animals; *P < 0.05 compared to vehicle control; HPF, high power field.

We previously observed that TGFβ/Smad3 treatment enhanced Akt phosphorylation in rat aortic SMCs^32^. In a recent study, it was found that in rat SMCs oxidized LDL activated the PI3K-Akt-mTOR pathway, which was inhibited by resveratrol^34^. In addition, in our current study we found that LY249004, a commonly used inhibitor of the PI3K-Akt pathway, was able to block TGFβ/Smad3-stimulated KLF5 up-regulation (Fig. 6A). These observations together raise an interesting question as to whether TGFβ/Smad3 activated the Akt-mTOR pathway which in turn acted as the upstream regulator of KLF5 protein up-regulation and hence the resveratrol target. Therefore, after establishing that resveratrol inhibits TGFβ/Smad3-stimulated mTOR activation and KLF5 up-regulation, we determined the effect of resveratrol on Akt activation. We found that while agreeing with our previous report^32^ TGFβ/Smad3 increased phospho-Akt ~2 fold, resveratrol abolished this stimulation (Fig. 7A). In contrast, rapamycin did not show an effect on Akt phosphorylation, placing mTOR downstream of Akt activation. We then further determined whether resveratrol inhibited TGFβ/Smad3-stimulated Akt activation by blocking Smad3 phosphorylation. As shown in Fig. 7B, a lack of resveratrol effect on TGFβ-stimulated Smad3 phosphorylation ruled out this possibility. In sum, these results together tracked down the target of resveratrol inhibitory effect on TGFβ/Smad3-stimulated KLF5 protein up-regulation and SMC de-differentiation, to the Akt-mTOR pathway which was downstream of TGFβ/Smad3 in our experimental system.

After tracking down the resveratrol functional target to Akt phosphorylation, we finally evaluated this effect in balloon-injured arterial wall, where the TGFβ/Smad3 signaling is known to be elevated^21^. Using the artery sections from the periadventitial delivery experiments, we found that while p-Akt staining was enhanced in the injured arteries compared to uninjured control at 14 days after surgery, periadventitial treatment with resveratrol significantly reduced Akt phosphorylation (Fig. 7C). As the effect of resveratrol on p-Akt has not been previously examined in neointima-producing injured arteries, our data provide new information in regard to *in vivo* targets of resveratrol for potential pharmacological interventions to inhibit IH.

## Discussion

**Figure 8:**
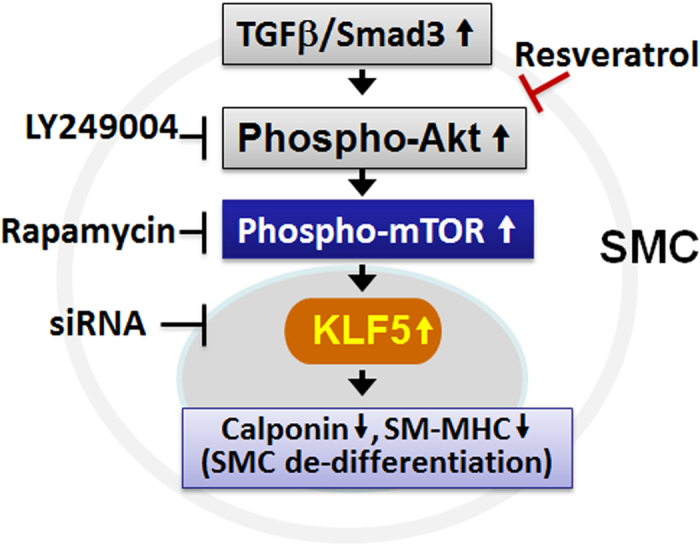
A schematic of resveratrol-targeted pathway in rat vascular smooth muscle cells.

Restenosis remains a major cause of failure of reconstructive vascular interventions. The failure rate is particularly high among the patients undergoing open surgical procedures including bypass, endarterectomy, and dialysis access, as there is no translated anti-restenotic method for these patients^18^. With this clinical need in mind, we carried out research with two chief objectives: 1) To evaluate periadventitial delivery as a viable approach to optimize the effects of resveratrol on all three major pro-restenotic pathologies. We observed that periadventitial delivery of resveratrol produced a markedly improved neointima-inhibiting effect versus previously reported systemic administration (86% versus ~30–60%^6^,^7^,^8^,^9^,^10^,^11^). Importantly, perivascular delivery of resveratrol did not retard, but rather accelerated post-surgery endothelial recovery and did not cause constrictive remodeling. 2) To investigate the molecular mechanism of resveratrol-promoted re-differentiation of SMCs using an *in vitro* model of TGFβ/Smad3 stimulation. We found that while TGFβ/Smad3 stimulated mTOR activation and up-regulated KLF5 protein — a transcription factor known to promote SMC de-differentiation and IH^28^, resveratrol abolished KLF5 up-regulation and re-differentiated SMCs by targeting the Akt-mTOR pathway downstream of TGFβ/Smad3 (Fig. 8). These findings suggest that resveratrol represents a promising candidate drug for an anti-restenotic perivascular delivery paradigm that is uniquely suited for open surgeries.

Three major pathologies contribute to restenosis: SMC hyperplasia in the intima (IH) due to SMC pathogenic phenotype transformation, impairment of re-endothelialization which exacerbates IH, and constrictive remodeling that shrinks the vessel diameter and lumen. An unfavorable effect on any of these three could compromise the long-term efficacy of an anti-restenotic therapy^5^. Current clinical methods of rapamycin or paclitaxel-eluting stents not only cause constrictive remodeling at stent edge^2^ but also impinge on the endothelium leading to thrombosis^4^. These inherent defects are increasingly recognized necessitating vigorous research on alternative therapeutics/paradigms. Recently, resveratrol has shown an anti-IH effect in several preclinical studies^6^,^7^,^8^,^9^,^10^,^11^. However, the overall outcome is not satisfactory. We noted that in these studies resveratrol was administered exclusively via systemic approaches, which are not optimal because natural compounds such as resveratrol are extremely susceptible to metabolic clearance when systemically delivered^13^. Even though resveratrol coated on a stent reduced stenosis in rat carotid artery by 60% 4 weeks after stenting, resveratrol on the stent is subject to loss to the circulation and its longer-term effect is not known^37^. We thus adopted a *periadventitial* approach to circumvent this problem. Moreover, our studies differ from the existing publications also in that we determined the resveratrol effects on all three major pro-restenotic pathologies as well as apoptosis in the vessel wall whereas none of the previous reports offered a full-spectrum analysis. Significantly, our data indicate that periadventitial delivery of resveratrol produced highly desirable effects on IH prevention and endothelium protection, and also favorably, no constrictive remodeling. Furthermore, another unique progress made in our study is that we also tested a 3-month long-term resveratrol treatment of injured rat carotid arteries using a PCL polymer sheath^18^, which produced a lasting inhibitory effect on IH without causing constrictive remodeling. Over a long term (months) of drug delivery multiple factors including the degradation products of the drug carrier would elicit inflammatory or mechanical stress on the vessel wall leading to constrictive remodeling. In this regard, the positive outcome from our 3-month periadventitial resveratrol treatment is promising and lays a basis for future optimization.

Improvement of resveratrol efficacy by using a periadventitial approach versus systemic delivery could be rationalized by the following. 1) Since resveratrol applied outside the artery does not directly enter the circulation, its bioavailability could be protected^15^. Resveratrol would be otherwise subjected to quick degradation in various organs if systemically delivered^12^,^14^. 2) Periadventitial local delivery may provide relatively high focal drug concentrations in pathogenic SMCs in the vessel wall. In support of these assertions, at 24 h after periadventitial application we still detected non-metabolized resveratrol as the major form in the vessel wall (Figure S2), whereas resveratrol in the circulation (serum) can be completely metabolized in less than 15 min^14^,^38^. Moreover, in a new report by Tolva *et al*.^14^, local delivery of a resveratrol-containing compound into injured rabbit arteries with a balloon catheter effectively preserved active forms of resveratrol in the arterial wall and reduced the intima/media area ratio by ~70% compared to sham control. 3) In addition, accelerated post-surgery endothelial recovery may also contribute to improved efficacy of periadventitial delivery of resveratrol. Unlike SMCs, ECs are highly susceptible to drug toxicity^4^,^36^. In a recent report using systemic delivery, whereas a low concentration of resveratrol promotes re-endothelialization after angioplasty, a high resveratrol concentration failed to produce this effect^6^. Indeed, high concentrations of resveratrol have been shown to induce apoptosis in several cell types^34^. Of note, periadventitial release could generate a gradient^15^,^16^ of resveratrol with higher concentrations in the adventitia and lower concentrations in the endothelium, and may have thus protected endothelial recovery. A protective effect of appropriate concentrations of resveratrol on ECs has been previously demonstrated *in vitro*^6^,^39^. While ECs are highly sensitive to oxidative and inflammatory damage, resveratrol produces antioxidant and anti-inflammatory effects^11^,^34^, or stimulates eNOS expression^6^, which is generally thought to be beneficial to ECs. Taken together, our *in vivo* results suggest that periadventitial delivery of resveratrol represents a viable strategy to optimally produce multi-beneficial effects of this promising anti-restenotic drug.

The functional mechanisms of resveratrol are complex. This complexity stems from the fact that resveratrol targets multiple proteins and pathways. To date four direct-binding targets of resveratrol have been discovered. Earlier sirtuin-1 was identified as a resveratrol target^40^. It was then reported that resveratrol is a PDE4 inhibitor which activates AMPK by lowering intracellular AMP concentrations^41^. Most recently, resveratrol was found to also directly bind to estrogen receptors^42^ and tyrosine transfer RNA synthetase^43^. Each of these resveratrol-binding sites is associated with numerous pathways, upstream and downstream. Therefore, it is impractical to delineate the resveratrol-associated mechanism in SMCs by investigating all the possible pathways. On the other hand, a growing body of evidence indicates that the therapeutic action of resveratrol is highly context-dependent. It may function by distinct mechanisms depending on cell type, the nature and duration of stimulation, and cell or disease state^12^. In order to minimize those confounding variables, we focused the resveratrol mechanistic studies specifically on vascular SMC de-differentiation using elevated TGFβ/Smad3 signaling as a stimulant, which is a known important contributor to IH^21^.

Built on our recent finding that TGFβ/Smad3 promote SMC de-differentiation^22^,^24^, herein we further uncovered that KLF5 plays a key role in this event. Up-regulation of KLF5 in SMCs by TGFβ/Smad3 has not been previously reported. Demonstrating a specific role of the TGFβ/Smad3 signaling axis in KLF5 regulation, TGFβ and AdSmad3 treatment each increased KLF5 protein and their combination further substantially enhanced KLF5 up-regulation. In addition, in our experimental system we did not observe a significant change of KLF5 protein levels after PDGF-BB treatment (data not shown), further supporting a specific role of TGFβ/Smad3 in KLF5 up-regulation. An ensuing question was whether TGFβ/Smad3 regulated KLF5 mRNA or protein, since Smad3 is a transcription factor that regulates the transcription of hundreds of genes. Surprisingly, our data did not show a significant difference in KLF5 mRNA levels between TGFβ/Smad3 stimulation and GFP control. We were thus able to narrow down the TGFβ/Smad3 regulation of KLF5 to the protein level. Inasmuch as both the TGFβ/Smad3 axis and KLF5 are proven important contributors to IH^21^,^27^, our finding of KLF5 regulation by TGFβ/Smad3 opens a new conduit for better understanding of neointimal pathogenesis and its mitigation.

Using resveratrol and rapamycin as powerful pharmacological tools, we further discovered that activation of mTOR is responsible for TGFβ/Smad3-stimulated up-regulation of KLF5. While resveratrol treatment abrogated TGFβ/Smad3-stimulated activation of mTOR and its downstream effector S6K, it also reversed TGFβ/Smad3-stimulated KLF5 up-regulation, suggesting mTOR as an upstream positive regulator of KLF5. Further confirming the role of mTOR in KLF5 regulation, treatment with rapamycin eliminated the increase of KLF5 protein caused by TGFβ/Smad3 stimulation. The up-regulation of KLF5 protein by activated mTOR observed herewith is a novel finding that has never been previously reported, neither in SMCs nor in other cell types. It is established that the central function of activated mTOR is promotion of protein translation, consistent with our data that KLF5 protein rather than mRNA was increased following TGFβ/Smad3-stimulated mTOR activation. Importantly, while KLF5 is a transcription factor known to promote SMC de-differentiation^28^, rapamycin inhibits SMC de-differentiation by targeting mTORC1^19^. Furthermore, Liu *et al*. noted that while rapamycin in SMCs up-regulated TET2, a pro-differentiation epigenetic factor, siRNA knockdown of TET2 increased de-differentiating factors including KLF5^19^. Although in their paper there is no data available for a direct effect of rapamycin on KLF5 protein levels, the stimulatory effect of rapamycin on TET2 and the inhibitory action of TET2 on KLF5 combined support a real possibility of an inhibitory effect of rapamycin on KLF5, as observed herewith. It is worth noting however, TET2 siRNA increased KLF5 mRNA but in our study TGFβ/Smad3 stimulated the production of KLF5 protein but not mRNA. It is therefore premature to propose that TGFβ/Smad3-stimulated mTOR activation enhanced KLF5 protein production via TET2 down-regulation. Rather, in our experimental setting, activated mTOR likely up-regulates KLF5 protein by a distinct mechanism, which is our next research subject.

Our finding of positive regulation of KLF5 protein by mTOR is significant especially considering that mTOR and KLF5 both are crucial regulators in SMC pathophysiology and vascular disease. mTOR is a master regulator with influence on almost every cellular activity. In complex with protein partners, this atypical kinase senses and integrates diverse environmental cues including growth factors, and couples these signals to promotion of protein translation and lipid synthesis. A critical role of mTOR in recurrent vascular disease is best demonstrated by the clinical use of rapamycin as an anti-restenotic drug (on stents) that potently blocks SMC proliferation and migration^19^. Moreover, as reported by Liu *et al*., rapamycin also inhibits SMC de-differentiation, and this function is likely related to KLF4 and KLF5^19^.

With regard to a role in SMC de-differentiation, KLF4 has been at the center of interests as well as debates, partly because it is one of the four Yamanaka factors essential for reprogramming differentiated cells to pluripotency^26^. Whereas some studies show that KLF4 is a potent stimulator of SMC de-differentiation^25^,^35^, there are also reports indicating an opposite KLF4 function^44^. Further confounding evidence was from *in vivo* studies. As demonstrated by at least two independent groups, knockout or knockdown of KLF4 did not mitigate but rather exacerbated IH after arterial injury^35^. Unlike KLF4, KLF5 has been shown to promote SMC de-differentiation, proliferation and migration *in vitro* consistently by different research groups^27^,^28^. As for the mechanism, a plausible explanation is that KLF5 competes with myocardin for binding with the transcription factor SRF thus suppressing pro-differentiation genes in SMCs^28^. Importantly, while overexpression of KLF5 enhanced, knockdown of KLF5 reduced IH in a rat balloon angioplasty model^27^. Moreover, application of miR145, which down-regulated its target protein KLF5, reduced IH as well^45^. Taken together these solid studies and our own results, the mTOR/KLF5 axis appears to be an important player in SMC phenotype transformation, in particular, in TGFβ/Smad3-initiated SMC de-differentiation. Significantly, resveratrol provides an effective inhibitor alternative to rapamycin in curbing IH-promoting SMC pathophysiology.

In pursuit of the regulators upstream of the mTOR/KLF5 axis and downstream of TGFβ/Smad3, we focused our attention on the well-established Akt-mTOR pathway. While we previously showed that TGFβ/Smad3 stimulated Akt phosphorylation via direct protein interactions^32^, Brito *et al*. reported that resveratrol inhibited the Akt-mTOR pathway in SMCs *in vitro*^34^. It is important to note that although our results agree with Brito *et al*. on the resveratrol inhibition of the Akt-mTOR pathway in SMCs, our studies differ in at least three areas: (1) We focused on SMC de-differentiation which was not addressed by Brito *et al*. (2). Whereas Brito *et al*. used ox-LDL to stimulate SMC proliferation^34^, we used TGFβ/Smad3 to de-differentiate SMCs. (3) Most importantly, our *in vitro* experiments identified mTOR/KLF5 as a novel regulatory axis mediating TGFβ/Smad3-stimulated SMC de-differentiation. Nonetheless, both studies support the important role of resveratrol as an inhibitor of the Akt-mTOR pathway. Furthermore, an inhibitory effect of resveratrol on TGFβ/Smad3-stimulated Akt activation and a lack of resveratrol effect on Smad3 activation together confine the target of resveratrol to the downstream of Smad3 and upstream of Akt (Fig. 7C). Although there is evidence that resveratrol targets PI3K, it remains unclear whether resveratrol binds directly to this kinase^34^.

It was recently reported that at a concentration ≥25 μM, resveratrol activates AMPKα (phosphorylation at T172) and inhibits Akt in cardiac myocytes^46^ as well as in human^20^ and rat^34^ vascular SMCs. Moreover, Thompson *et al*. reported that resveratrol inhibits human SMC de-differentiation via AMPK activation^20^. We therefore asked the question whether in our study resveratrol influenced TGFβ/Smad3-sitmulated signaling via AMPKα phosphorylation in rat SMCs. Our Western blot assays indicate that stimulation of rat SMCs with TGFβ/Smad3 did not alter levels of phosphorylated AMPKα(T172) at either of the six time points from 15 min to 24 h (data not shown). In accordance to our observations, Brito *et al*. reported that ox-LDL, another stimulant, did not change phospho-AMPKα(T172) either, and they concluded that resveratrol inhibited the Akt-mTOR pathway independent of AMPK activation^34^. In addition, a report using fibroblasts indicates that resveratrol inhibits mTOR signaling in an AMPK- independent manner^47^. Taken together, our results suggest a mechanism underlying the effective resveratrol inhibition of TGFβ/Smad3-stimulated SMC de-differentiation, i.e., resveratrol abrogates KLF5 protein up-regulation by blocking the activation of the Akt-mTOR pathway, as summarized in Fig. 8.

## Conclusions

Through systematic and longitudinal *in vivo* analysis we find that periadventitial delivery of resveratrol produces highly desirable anti-restenotic outcomes. Two weeks after balloon injury of rat carotid arteries, neointima was nearly eliminated, endothelium recovery was accelerated, and constrictive remodeling was avoided. This therapeutic effect is significant especially considering a paucity of publications reporting anti-restenotic methods (or agents) with favorable effects on mitigating all three major pro-restenotic pathologies. Furthermore, 3 months of periadventitial treatment with resveratrol delivered by a polymer sheath produced lasting suppression of neointima growth without a side effect of constrictive remodeling. Given that restenosis in human patients is a chronic process, while still rare, demonstration of long-term efficacy of endothelium-protective anti-restenotic methods is highly desirable. This is particularly true for resveratrol, a promising anti-restenotic drug but fretting researchers for its extremely low bioavailability^14^. Furthermore, our *in vitro* studies using resveratrol as a pharmacological probe identified a novel regulation; i.e., while elevated TGFβ/Smad3 signaling de-differentiate SMCs by up-regulating transcription factor KLF5, resveratrol blocks KLF5 up-regulation and re-differentiates these activated SMCs by disrupting the Akt-mTOR pathway. Inasmuch as there is no clinical method available to date for the prevention of restenosis following open surgery, periadventitial delivery of resveratrol would potentially lead to an effective paradigm to meet this urgent clinical need, in thousands of patients who are subject to frequent failure of open surgical interventions.

## Additional Information

**How to cite this article:** Zhu, Y. *et al*. Restenosis Inhibition and Re-differentiation of TGFβ/Smad3-activated Smooth Muscle Cells by Resveratrol. *Sci. Rep.* **7**, 41916; doi: 10.1038/srep41916 (2017).

**Publisher's note:** Springer Nature remains neutral with regard to jurisdictional claims in published maps and institutional affiliations.

## Supplementary Material

Supplementary Figures
