# Supplementary material for: Restenosis Inhibition and Re-differentiation of TGFβ/Smad3-activated Smooth Muscle Cells by Resveratrol
Source: Sci Rep. 2017 Feb 6;7:41916. doi: 10.1038/srep41916 (PMC5292946; doi:10.1038/srep41916)
Supplement: Supplementary Figures [file srep41916-s1.doc]

**Restenosis Inhibition and Re-differentiation of TGFβ/Smad3-activated** **Smooth Muscle Cells by Resveratrol**

*Short title: Periadventitial Delivery of Resveratrol*

Yichen Zhu**^1,3,#^**, Toshio Takayama**^2,#^**, Bowen Wang**^1, #^**, Alycia Kent**^1^**; Mengxue Zhang**^1^**, Bernard Y.K. Binder**^4^**, Go Urabe**^1^**, Yatao Shi**^5^**, Daniel DiRenzo**^1^**, Shakti A. Goel**^1^**, Yifan Zhou**^1^**, Christopher Little**^2^**, Drew A. Roenneburg**^2^**, Xu Dong Shi**^1^**, Lingjun Li**^5,6^**, William L. Murphy**^4^**, K. Craig Kent**^1,2^**, Jianjuan Ke**^7^,** *, Lian-Wang Guo**^1^,** *

**^1^**Department of Surgery, University of Wisconsin, 5151 Wisconsin Institute for Medical Research, 1111 Highland Ave, Madison, WI 53705, U.S.A.

**^2^**University of Wisconsin Hospital and Clinics, 600 Highland Ave, Madison, WI 53792, U.S.A.

**^3^**Department of Urology, Capital Medical University Beijing Friendship Hospital, N0.95, Yong`an Road, Xicheng district, Beijing, China

**^4^**Department of Biomedical Engineering, University of Wisconsin, 5009 Wisconsin Institute for Medical Research, 1111 Highland Ave, Madison, WI 53705, U.S.A.

**^5^**School of Pharmacy and Department of Chemistry, University of Wisconsin, Madison, WI 53705, U.S.A.

^6^School of Life Sciences, Tianjin University, No.92 Weijin Road, Nankai District, Tianjin 300072, China

**^7^**Department of Anesthesiology, Medical College, Wuhan University, 169 Donghu Road, Wuhan, Hubei, PR China

**^#^**These authors contributed equally to this work.

* Corresponding authors

Lian-Wang Guo, PhD

Department of Surgery, University of Wisconsin

1111 Highland Avenue, WIMR 5151
Madison, WI 53705

Tel.: +1 608 262 6269. Fax: +1 608 262 3330.

Email: guo@surgery.wisc.edu

Jianjuan Ke, MD, PhD

Department of Anesthesiology

Medical College, Wuhan University

169 Donghu Road, Wuhan, Hubei, PR China

Tel: +86-13971687409

Email: [kejianjuan2009@163.com](mailto:kejianjuan2009@163.com)

The authors declare that no conflict of interest exists.

**Supplemental figures**

***
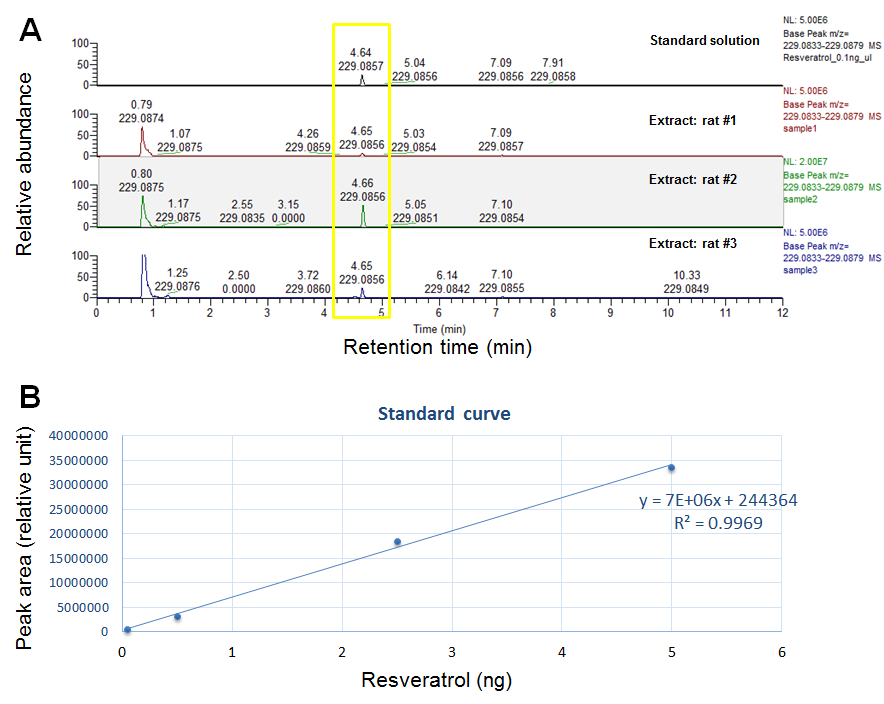
***

***Figure S1. MS detection of resveratrol in rat common carotid artery tissue homogenates***

Injured and resveratrol-treated common carotid arteries were collected from 3 rats 24 hours after angioplasty injury. Resveratrol extracts from the tissue homogenates were used for MS analysis, as described in detail in Methods. (A) MS spectra of a resveratrol standard solution and extracts from 3 rats. The peak of m/z 229.0857 with a retention time of 4.64 min detected in the standard solution is identified as the protonated ion of resveratrol. Based on this data, the ions of m/z 229.0856 with a retention time of 4.65 min or 4.66 min (boxed peaks) represent protonated resveratrol in tissue extracts. (B) Standard curve of peak area plotted against nanograms of resveratrol showing a linear range of MS signal in response to increasing concentrations of resveratrol.

***
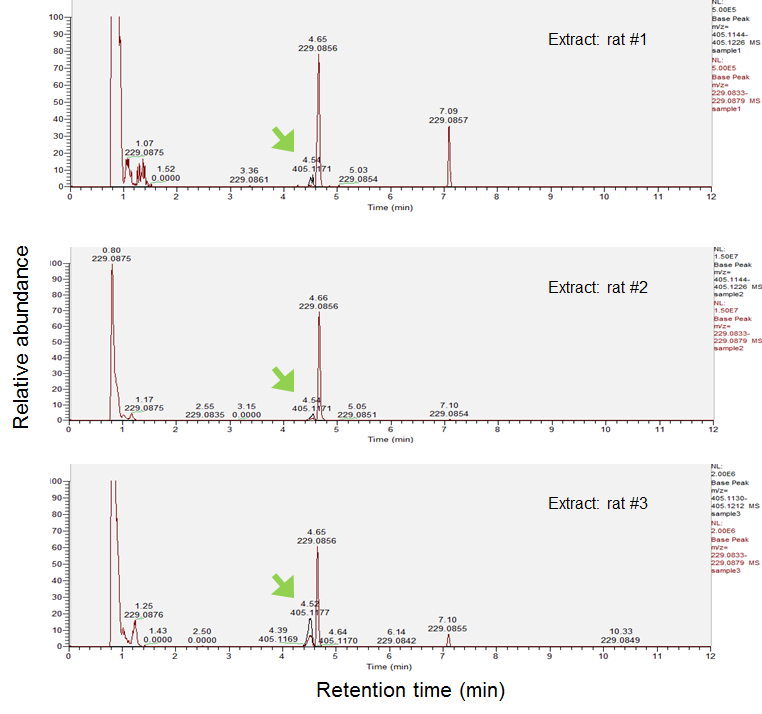
***

***Figure S2. MS detection of resveratrol metabolite(s) in rat common carotid artery tissue homogenates***

Shown are enlarged versions of the corresponding spectra in Figure S1. The peak of m/z 405.1171 (arrow) with a retention time of 4.54 min represents the protonated ion of resveratrol glucuronide with mass error below 5 ppm. Accordingly, the resveratrol glucuronide/resveratrol ratios in artery tissue samples of 3 rats are calculated to be 10.8%, 11.3% and 63%, respectively.
